# Phenformin has anti-tumorigenic effects in human ovarian cancer cells and in an orthotopic mouse model of serous ovarian cancer

**DOI:** 10.18632/oncotarget.22012

**Published:** 2017-10-24

**Authors:** Amanda L. Jackson, Wenchuan Sun, Joshua Kilgore, Hui Guo, Ziwei Fang, Yajie Yin, Hannah M. Jones, Timothy P. Gilliam, Chunxiao Zhou, Victoria L. Bae-Jump

**Affiliations:** ^1^ Division of Gynecologic Oncology, University of Cincinnati, Cincinnati, OH, USA; ^2^ Division of Gynecologic Oncology, University of North Carolina at Chapel Hill, Chapel Hill, NC, USA; ^3^ Houston Methodist Gynecologic Oncology Associates, Houston, TX, USA; ^4^ Department of Gynecologic Oncology, Shandong Cancer Hospital & Institute, Jinan, P.R. China; ^5^ Department of Obstetrics, Beijing Obstetrics and Gynecology Hospital, Capital Medical University, Beijing, P.R. China; ^6^ Lineberger Comprehensive Cancer Center, University of North Carolina at Chapel Hill, Chapel Hill, NC, USA

**Keywords:** ovarian cancer, phenformin, AMPK/mTOR pathway, cell proliferation, apoptosis

## Abstract

Obesity and diabetes have been associated with increased risk and worse outcomes in ovarian cancer (OC). The biguanide metformin is used in the treatment of type 2 diabetes and is also believed to have anti-tumorigenic benefits. Metformin is highly hydrophilic and requires organic cation transporters (OCTs) for entry into human cells. Phenformin, another biguanide, was taken off the market due to an increased risk of lactic acidosis over metformin. However, phenformin is not reliant on transporters for cell entry; and thus, may have increased potency as both an anti-diabetic and anti-tumorigenic agent than metformin. Thus, our goal was to evaluate the effect of phenformin on established OC cell lines, primary cultures of human OC cells and in an orthotopic mouse model of high grade serous OC. In three OC cell lines, phenformin significantly inhibited cellular proliferation, induced cell cycle G1 arrest and apoptosis, caused cellular stress, inhibited adhesion and invasion, and activation of AMPK and inhibition of the mTOR pathway. Phenformin also exerted anti-proliferative effects in seven primary cell cultures of human OC. Lastly, phenformin inhibited tumor growth in an orthotopic mouse model of serous OC, coincident with decreased Ki-67 staining and phosphorylated-S6 expression and increased expression of caspase 3 and phosphorylated-AMPK. Our findings demonstrate that phenformin has anti-tumorigenic effects in OC as previously demonstrated by metformin but it is yet to be determined if it is superior to metformin for the potential treatment of this disease.

## INTRODUCTION

Ovarian cancer (OC) is one of the leading causes of cancer related deaths among women worldwide with an overall 5-year survival of only 30-40% [1]. Increasing evidence suggests that obesity is a significant risk factor for OC and is associated with worse outcomes for this disease [2–9]. Obesity has reached epidemic proportions in the United States, with over 30% of adults considered obese and 65% considered overweight based on their body mass index (BMI) [10]. Impaired glucose regulation and insulin resistance are consequences of obesity, often culminating in Type II diabetes. Type II diabetes in addition to obesity appears to affect OC survival. A recent study of 642 women with OC over a 10-year period found a significantly worse overall survival in diabetics as compared to non-diabetics, even after multivariable adjustment [11].

Overweight and obese states may be linked to OC through nutrient sensitive signaling cascades, such as the insulin/insulin growth factor (IGF) and PI3K/Akt/mTOR pathways [12]. Hyperinsulinemia, IGF-1, and IGF-1 receptor (IGF-1R) levels are known to be important in OC development and progression [13–15]. Signaling through IGF-1R leads to activation of the downstream PI3K/Akt/mTOR pathway, and components of this pathway are often mutated, amplified or aberrantly expressed in OCs [16, 17]. In addition, our previous work found that the metabolic effects of obesity promoted OC progression and aggressiveness in a genetically engineered mouse model of high grade serous OC [18]. Therefore, a metabolic approach to the treatment of OC may provide a novel strategy to improve outcomes for this invariably lethal disease.

Metformin, a biguanide drug, is one of the most widely prescribed treatments for Type II diabetes. Epidemiological evidence suggests that metformin lowers cancer risk and reduces cancer incidence and deaths among diabetic patients [19–21], including OC [22–26]. Pre-clinical studies have reported that metformin causes disruption of mitochondrial respiration leading to activation of AMP-activated protein kinase (AMPK) and inhibition of the mTOR pathway, ultimately decreasing cell proliferation, adhesion, migration and angiogenesis in OC cell lines and animal models [27–36]. Currently, metformin is being investigated in greater than 50 phase I, II and III clinical trials in a number of different cancers, including OC [37].

Phenformin is another biguanide with anti-diabetic activity. Phenformin is almost 50 times as potent as metformin for the treatment of diabetes, but it was withdrawn from the market in the United States in 1977 due to a small increased risk of lactic acidosis (64 cases per 100,000 patient-years), higher than that seen with metformin (3 in 100,000 years) [38]. Metformin is highly hydrophilic with a net positive charge at all physiologic pH values. Therefore, it requires cation-selective transporter proteins that mediate its entry into cells, including OCT1-3, PMAT and MATE 1-2 [39]. Phenformin is more lipophilic and is not reliant on these transporter proteins, allowing for higher concentrations of this biguanide over metformin to accumulate intracellularly. It has been theorized that phenformin may have heightened anti-tumorigenic efficacy as compared to metformin, due to increased uptake of this drug by tumor cells. *In vitro* and *in vivo* studies in breast, prostate, lung, melanoma, glioblastoma and colon cancer demonstrate that phenformin is more potent for inhibiting cell proliferation and tumor growth than metformin [40–46]. Compared to metformin, phenformin has also been shown to be a more potent inhibitor of mitochondrial complex I, which causes overproduction of reactive oxygen species [47]. Thus, our objective was to evaluate the potential anti-tumorigenic effects of phenformin in OC cell lines, primary cultures of human OC cells and in an orthotopic mouse model of high grade serous OC. Our hypothesis was that phenformin would demonstrate anti-tumorgenic effects similar to metformin in OC. Our results showed that phenformin is a promising agent to inhibit cell proliferation and tumor growth in OC cell lines, primary cultures of human OC and in an orthotopic mouse model of high grade serous OC.

## RESULTS

### Phenformin inhibits cell proliferation in OC cells

**Figure 1:**
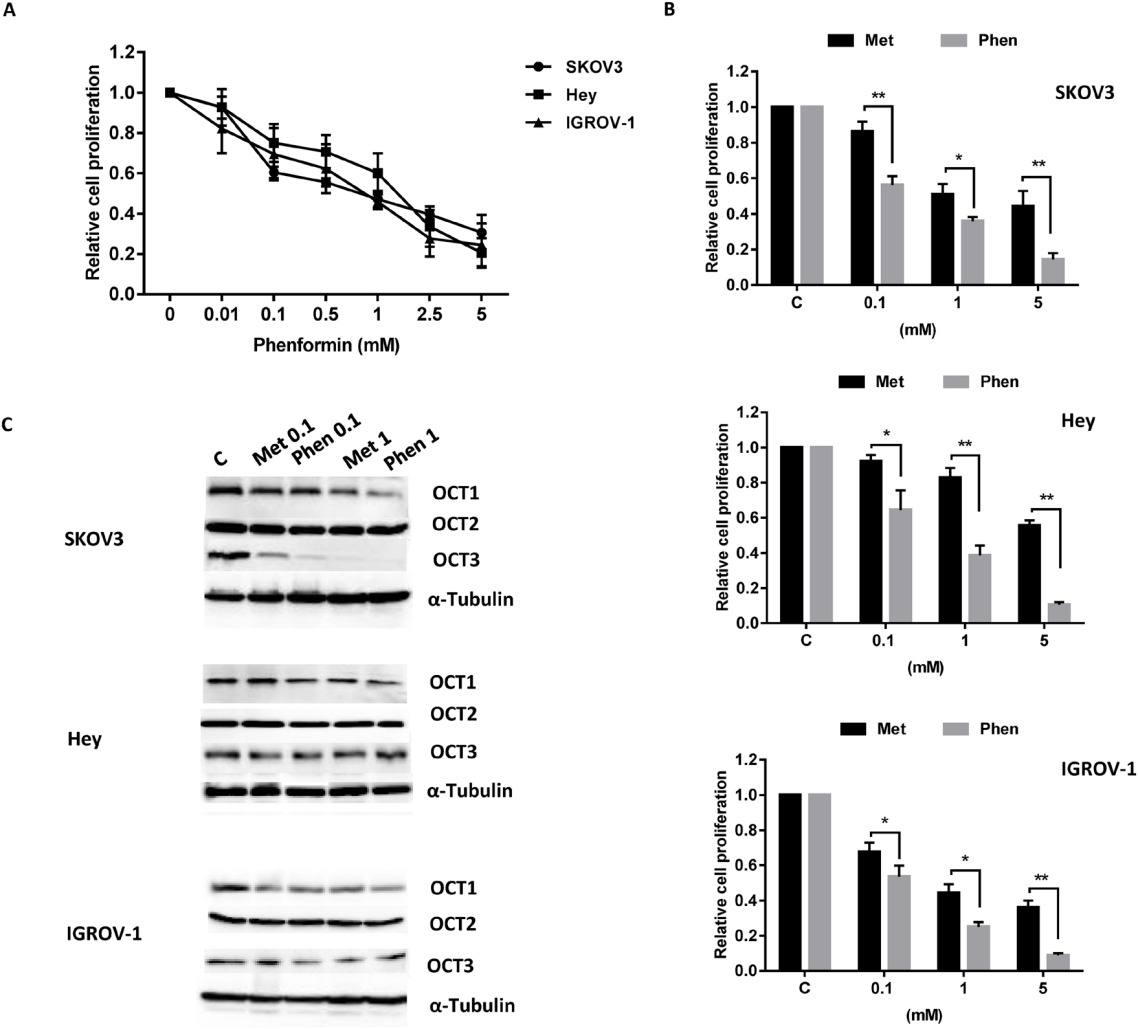
Effect of phenformin on cell proliferation in the OC cells The SKOV3, Hey and IGROV-1 cell lines were cultured in the presence of varying concentrations of phenformin for 72 hours. Cell proliferation was determined by MTT assay. **(A)** The comparison of metformin and phenformin in inhibition of cell proliferation was assessed after 72 hours of treatment. **(B)** The effect of metformin and phenformin on OCT1, OCT2 and OCT3/4 on ovarian cancer cells was assayed by western blotting. **(C)** The results are shown as the mean ± SE of triplicate samples and are representative of three independent experiments.

The effect of phenformin on OC cell proliferation was assessed by MTT assay. Three OC cell lines, SKOV3, Hey and IGROV-1, were treated with varying concentrations of phenformin for 72 hours. As shown in Figure 1A, phenformin inhibited cell proliferation of all three OC cell lines in a dose-dependent manner after 72 hours of treatment. The mean IC50 value for SKOV3, Hey and IGROV-1 was 0.9, 1.75 and 0.8 mM, respectively. These results suggest that phenformin effectively inhibits cell proliferation in OC cells. Successively, we compared the effect of phenfornin and metformin on cell proliferation in all three OC cell lines. We observed that both phenformin and metformin exhibited inhibitory effects on cell proliferation after 72 hours of treatment. However, phenformin at either low or high dosages was found to significantly increase the growth inhibition in the OC cell lines compared to metformin (Figure 1B). These results suggest that phenformin appeared more potent than metformin in inhibition of cell proliferation.

Given that biguanide is transported into cells by organic cation transporters (OCTs) 1, 2, and 3, we next investigated the affinity of phenformin and metformin for OCT1, OCT2 and OCT3/4 in the SKOV3, Hey and IGROV-1 cells. We treated three cell lines with 0.1 and 1 mM phenformin and metformin for 24 hours, respectively. Either metformin or phenformin decreased OCT1 and OCT3/4 expression in the OC cells, with the greatest effects seen in three cell lines after exposure to 1 mM phenformin. Phenformin and metformin did not affect OCT2 expression in all three cell lines (Figure 1C). These results when compared to metformin demonstrate that phenoformin may have enhanced affinity for OCT1 and OCT3 and improved potency over metformin in expression of OCT1 and OCT3.

### Phenformin induces cell cycle arrest and apoptosis

**Figure 2:**
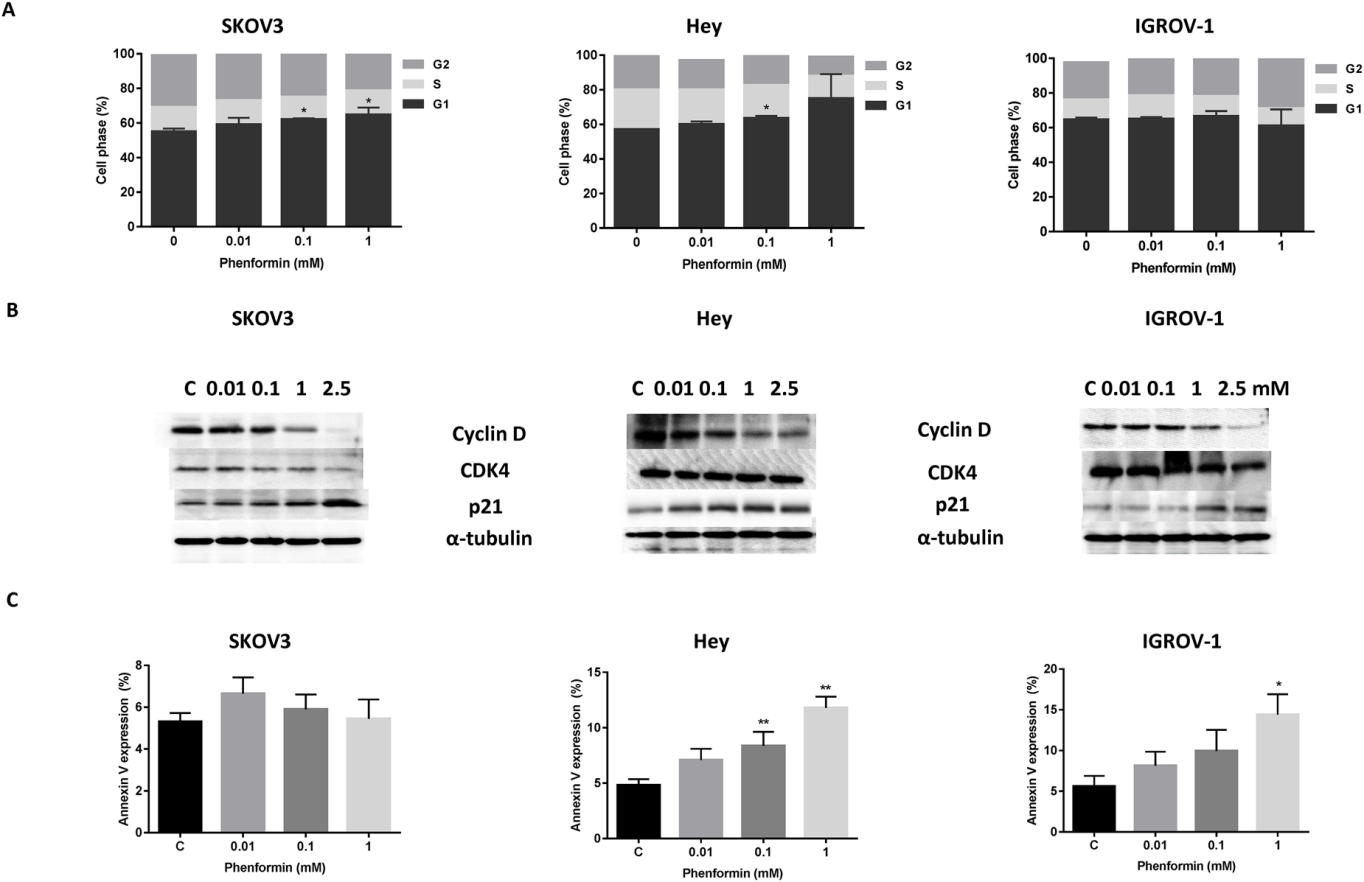
Effect phenformin on cell cycle progression and apoptosis in the OC cells The cells were treated with phenformin at different doses for 24 hours, and changes in cell cycle progression was analyzed by Cellometer. Phenformin induced G1 cell cycle arrest in the Hey and SKOV3 cell lines and G2 arrest in the IGROV-1 cell line. **(A)** Western blotting results showed that phenformin decreased CDK4 and cyclin D1 expression and increased p21 expression in all three cell lines. **(B)** The Hey, IGROV-1 and SKOV3 cells were grown for 24 hours and then treated with phenformin at the indicated concentrations for an additional 18 hours. Annexin V expression was detected by Cellometer. Phenformin induced apoptosis in the Hey and IGROV-1 cell lines, but not in the SKOV3 cell line. **(C)** The results are shown as the mean SD and are representative of three independent experiments.

To evaluate the underlying mechanism of growth inhibition by phenformin, the cell cycle profile was analyzed after treating the SKOV3, Hey and IGROV-1 cell lines with varying doses (0.01-2.5 mM) of phenformin for 24 hours. As illustrated in Figure 2A, phenformin induced G0/G1 cell cycle arrest and reduced S phase in the Hey and SKOV3 OC cell lines and increased G2 phase in the IGROV-1 OC cell line in a dose-dependent manner. These results suggest that phenformin induced cell cycle arrest through different checkpoints among the three OC cell lines.

To further characterize phenformin’s effect on cell cycle arrest, cell cycle-related proteins were analyzed in the phenformin-treated SKOV3, Hey and IGROV-1 cell lines. Western immunoblotting showed that phenformin down-regulated the cell cycle related proteins, cyclin D and CDK4 and increased p21 expression (Figure 2B). The effect of phenformin on apoptosis was evaluated using the Annexin V assay. This assay detects apoptotic cells by monitoring fluorescently labeled Annexin V, which binds to phosphatidylserine externalized on the surface of the cell membrane and is representative of a distinct phenomenon of early apoptosis. All cell lines were treated with phenformin at varying concentrations (0.01-1 mM) for 18 hours. The percentage of apoptotic cells increased distinctly in a dose-dependent manner in the Hey and IGROV-1 cell lines whereas phenformin had no effect on annexin V expression in the SKOV3 cells (Figure 2C). These results suggest that phenformin inhibits cell proliferation through divergent mechanisms in OC cells.

### Effect of phenformin on the AMPK/mTOR pathway

**Figure 3:**
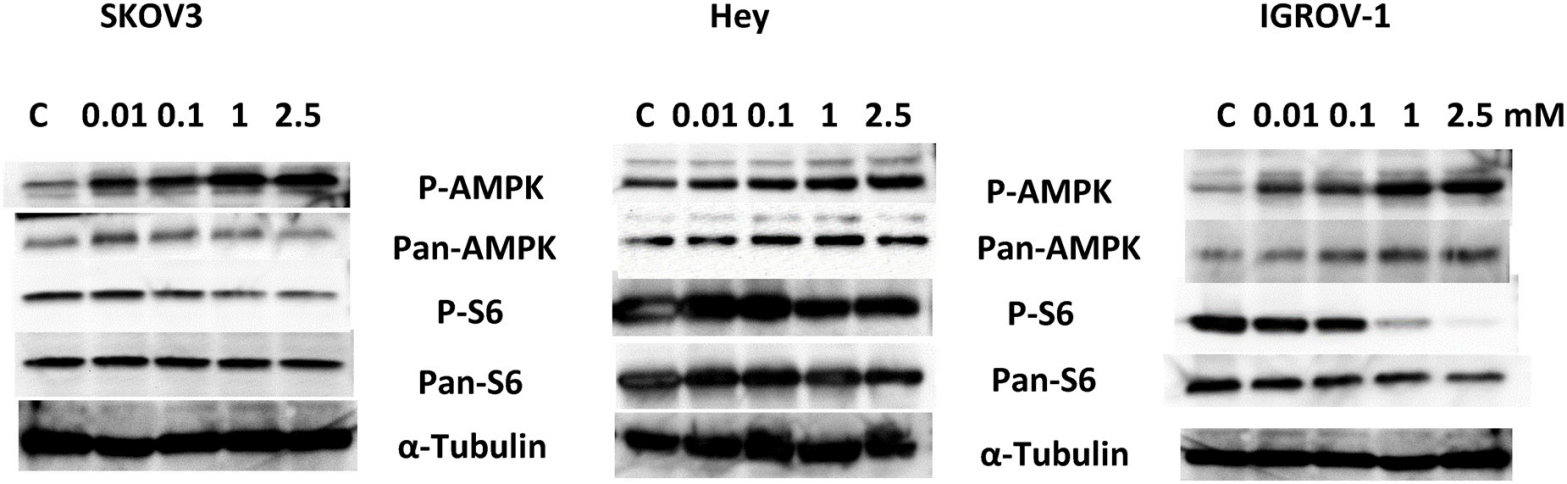
Phenformin activated AMPK and inhibited downstream targets of the mTOR pathway in the OC cells The Hey, IGROV-1 and SKOV3 cells were treated with phenformin at different doses for 24 hours. Western immunoblotting was used to assess the effect of phenformin on AMPK and the mTOR pathways. A dose-dependent reduction in phosphorylated-S6 and an increase in phosphorylated-AMPK protein levels was seen for all three cell lines, after 24 hours of exposure. Each experiment was performed twice to assess for consistency of results.

It is well known that AMPK and the mTOR pathway play a crucial role in the control of cell growth survival in OC, and targeting of these pathways leads to the inhibition of OC growth [48]. To investigate the mechanisms underlying the inhibition of ovarian cell growth by phenformin, we characterized the effect of phenformin on its immediate downstream signaling target, AMPK. Previous studies suggest that p70S6K is a downstream target of the mTOR pathway [49]. p70S6K kinase directly phosphorylates the 40S ribosomal protein S6, which results in enhanced translation of proteins that contain a polypyrimidine tract in the 5’-untranslated region [49]. Therefore, we studied the effect of phenformin on the phosphorylation of the S6 ribosomal protein and AMPK in all three cell lines. As expected, phenformin increased phosphorylation of AMPK and decreased phosphorylation of S6 in all three OC cell lines, within 24 hours of exposure (Figure 3). In addition, pan-S6 was decreased by phenformin in all three OC cell lines. Expression of pan-AMPK in the SKOV3 cells was not affected by phenformin.; however, an increase in pan-AMPK was seen in the Hey and IGROV-1 cell lines. This suggests that phenformin may exert its anti-proliferative effects through activation of AMPK and subsequent decreased phosphorylation of the S6 protein, resulting in inhibition of the mTOR pathway.

### Phenformin induces cellular stress in ovarian cancer cells

**Figure 4:**
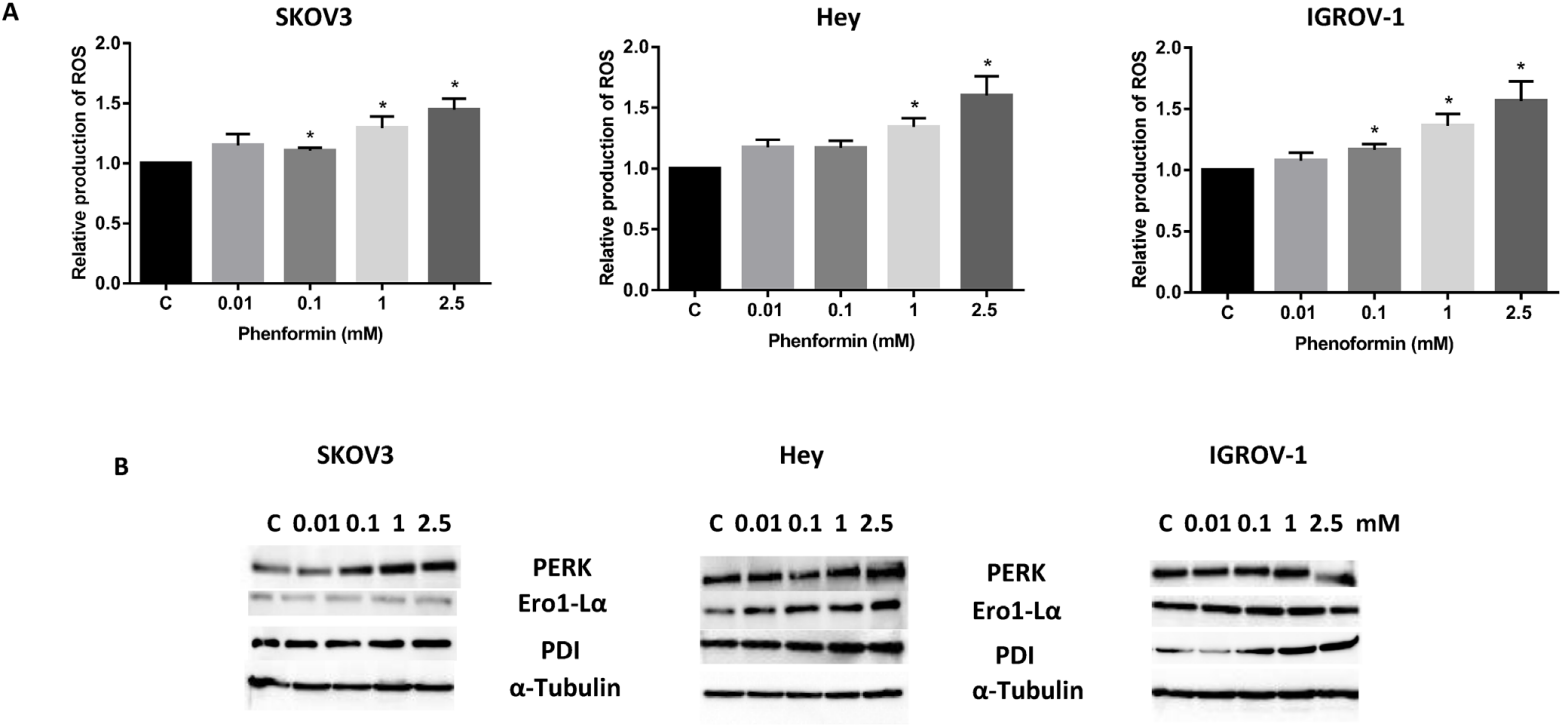
Phenformin induced cellular stress in ovarian cancer cells The Hey, IGROV-1 and SKOV3 cells were treated with phenformin at the indicated doses for 24 hours, and the ROS production was determined using the DCFH-DA assay. Phenformin increased the ROS level in a dose dependent manner. **(A)** The expression of cellular stress proteins (PERK, Ero1-1α, and PDI) was detected by western blotting after treatment of Phenformin for 24 hours. **(B)** ^*^p < 0.05 and ^**^p < 0.01.

Reactive oxygen species (ROS) have long been known to be a component of the cellular response to stress and production of ROS by diverse anti-cancer drugs has been closely related with the induction of apoptosis in cancers. Metformin has been shown to induce cell stress in different types of cancer [50]. To investigate the involvement of oxidative stress in the anti-proliferative effect of phenformin, intracellular ROS levels were examined using the ROS fluorescence indicator DCF-DA. Phenformin (0.01-2.5 mM) significantly increased ROS production in a dose-dependent manner in the OC cells after 24 hours of treatment (Figure 4A). We next examined the alternations of endoplasmic reticulum (ER) stress-related markers after treatment of phenoformin for 24 hours in the OC cells. Western blotting results showed that phenformin significantly induced the protein expression of PERK, Ero1-Lα and PDI in a dose dependent manner (Figure 4B). These results indicate that an increase in ROS production and ER stress might also be involved in the anti-tumorigenic effects of phenformin in ovarian cancer cells.

### Phenformin inhibits cell adhesion and invasion in ovarian cancer cells

**Figure 5:**
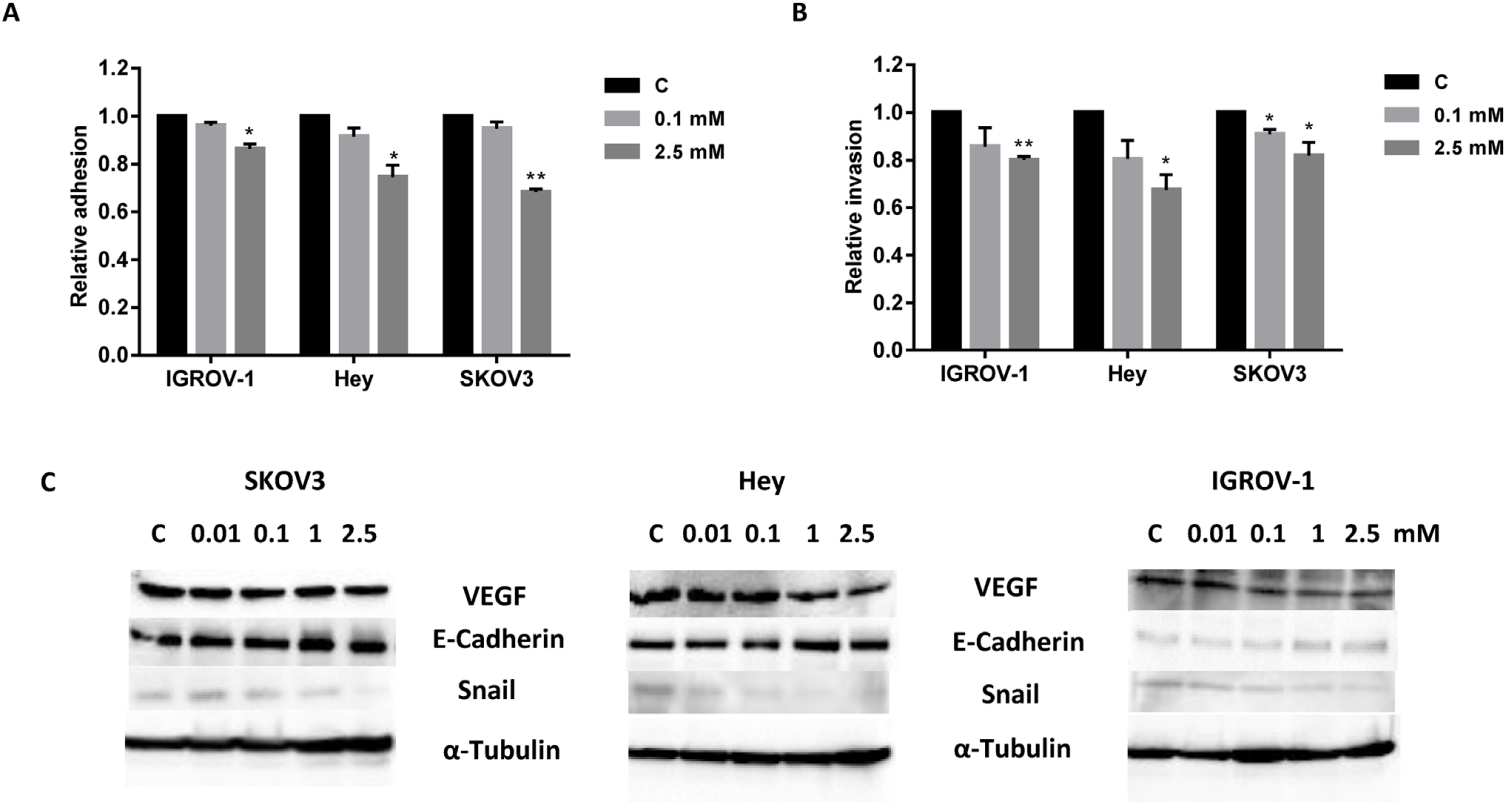
Phenformin blocked cell adhesion and invasion in ovarian cancer cells The Hey, IGROV-1 and SKOV3 cells were cultured for 24 hours and then treated with phenformin (0.01-2.5 mM) in a laminin coated 96 well plate or BME coated 96 transwell plate for 2 or 24 hours to assess adhesion and invasion in a plate reader. The data represents relative inhibition in each cell line. **(A** and **B)** The expression of VEGF, E–cadherin, and Snail were analyzed by Western blotting. **(C)** ^*^p < 0.05 and ^**^p < 0.01.

Adhesion and invasion are important steps leading to metastasis in ovarian cancer. In order to determine the effect of phenformin on adhesion and invasion of ovarian cancer cells, an *in vitro* laminin adhesion assay and transwell invasion system were employed, respectively. Incubation of the OC cell lines with phenformin at 0.1 and 2.5 mM for 2 hours showed a significant reduction in adhesion ( IGROV-1 in 5-17%, Hey cells in 9-23% and SKOV3 cells in 6-34%, p<0.05) (Figure 5A). Phenformin significantly blocked cell invasion after 24 hours of treatment in the OC cell lines (IGROV-1 in 17-25%, Hey cells in 20-29% and SKOV3 cells in 10-18%, p<0.05), as determined by the transwell invasion assay (Figure 5B). Inhibition of cell adhesion and invasion was dose-dependent in all three cell lines. To further analyze the effect of phenformin on motility and migration of ovarian cancer cells, the levels of expression of E–cadherin, Snail and VEGF were analyzed by Western blotting. After 24 hours of treatment, phenformin increased expression of E-cadherin and decreased expression of snail and VEGF (Figure 5C). Collectively, these results demonstrate that phenformin inhibits the adhesion and invasion of ovarian cancer cells.

### Phenformin decreases tumor growth in an orthotopic mouse model of serous OC

**Figure 6:**
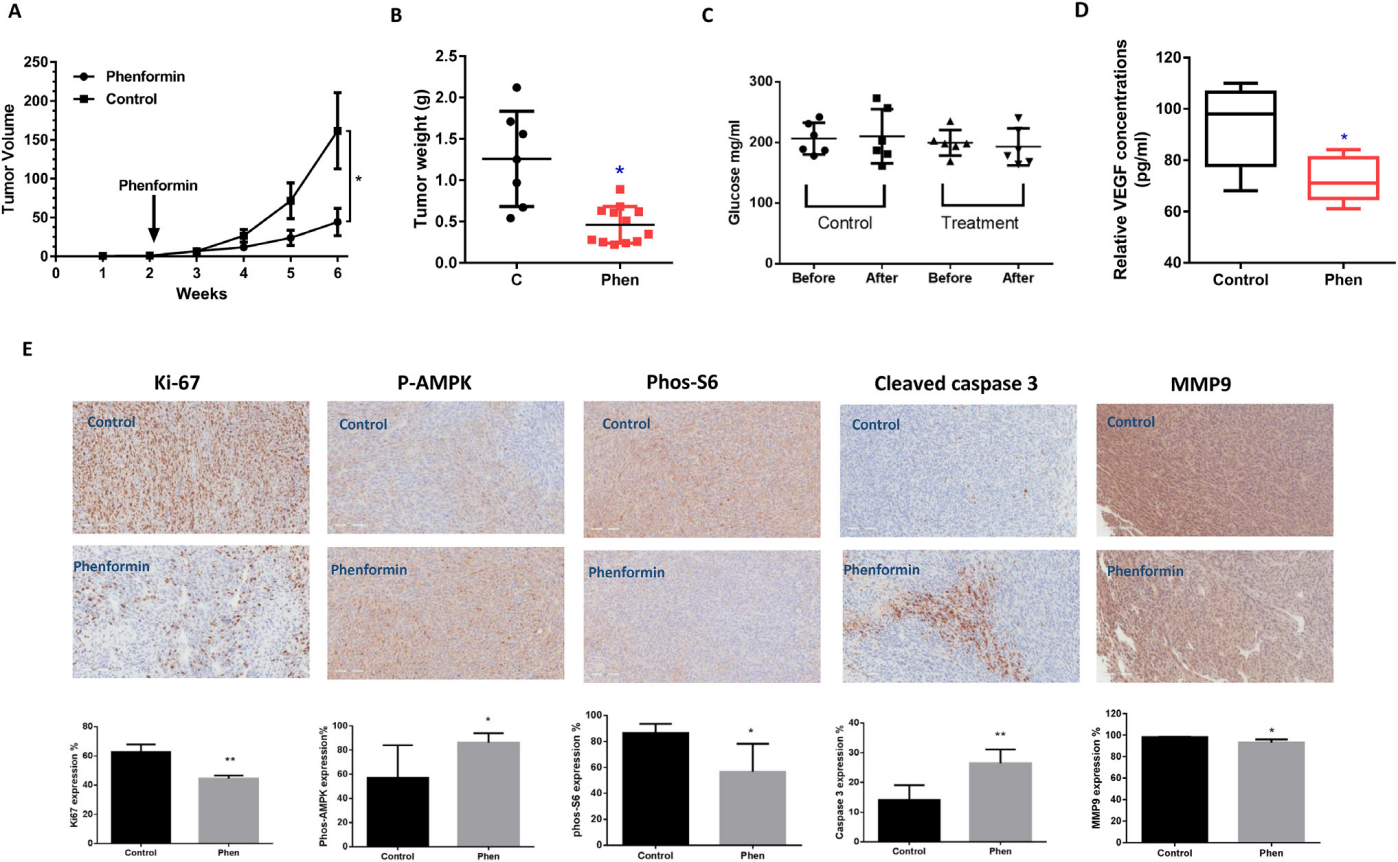
Phenformin reduced tumor growth of orthotropic xenografts of serous OC M909 cells were injected into left side of the ovarian bursa of 6–8 week old mice. When the tumors reached 0.1 cm in size (approximately 12 days after injection), the mice were treated with vehicle (control) or phenformin (2 mg/kg) once a day for 4 weeks. Tumor volume **(A)** and weight **(B)** were recorded during and after 4 weeks of treatment. Phenformin significantly inhibited tumor weight and volume in the M909 mice (n=13 animals per group) by 64% and 68%, respectively as compared to the vehicle treated controls (p=0.01014). Phenformin did not affect serum glucose levels in the mice. **(C)** Changes in Ki-67, phosphorylated-AMPK, phosphorylated-S6, MMP9 and cleaved caspase 3 with phenformin treatment were assessed by immunohistochemistry in the ovarian tumor tissues. **(D** and **E)** Phenformin decreased Ki-67. MMP9, and phosphorylated-S6 and increased caspase 3 and phosphorylated-AMPK in tumor tissues. ^*^p<0.05; ^**^p<0.01.

To validate the anti-tumorigenic potential of phenformin *in vivo*, we utilized an orthotopic high grade serous OC mouse model (M909) by injecting M909 cells into the ovary bursa of female mice [23]. When tumors reached a size of 0.1 cm, mice were treated with phenformin (2 mg/kg/day, intraperitoneal) or vehicle (saline) for 4 weeks. Tumor growth during the treatment was monitored by palpation and calipers twice a week. After 4 weeks of treatment, the mice were euthanized, and the ovarian tumors were removed, photographed, and weighed. Phenformin significantly inhibited tumor weight and volume in the M909 mice (n=13 animals per group) by 64% and 68%, respectively as compared to the vehicle treated controls (p=0.01014, Figure 6A and 6B). During the treatment, the mice showed tolerance to phenformin and maintained normal activities. Regular weekly measurements yielded no significant changes in blood glucose (Figure 6C) or body weight (data not shown). Serum levels of VEGF were found to be decreased significantly by 23% in the M909 mice treated with 4 weeks of phenformin compared to controls (Figure 6D).

To further confirm the anti-tumorigenic activity of phenformin *in vivo*, the expression of Ki-67, cleaved caspase 3, VEGF, phosphorylated-AMPK and phosphorylated-S6 in the ovarian tumor tissues was evaluated by immunohistochemistry (Figure 6E). Ki-67 was significantly reduced following phenformin treatment compared to the controls, whereas phenformin increased the levels of cleaved caspase 3 in the treated mice. Consistent with our results *in vitro*, the expression of phosphorylated-AMPK was increased and phosphorylated-S6 was decreased in the mice treated with phenformin as compared to untreated mice. In addition, we also found that phenformin significantly reduced the expression of VEGF in the M909 mice, suggesting that phenformin is able to inhibit cell invasion *in vivo*. These findings indicate that phenformin, like other biguanides, inhibits OC growth *in vivo* via activation of AMPK and inhibition of the mTOR pathway, both of which are critical signaling targets involved in proliferation and metabolism.

### Phenformin inhibited proliferation of OC cells derived from patients

**Figure 7:**
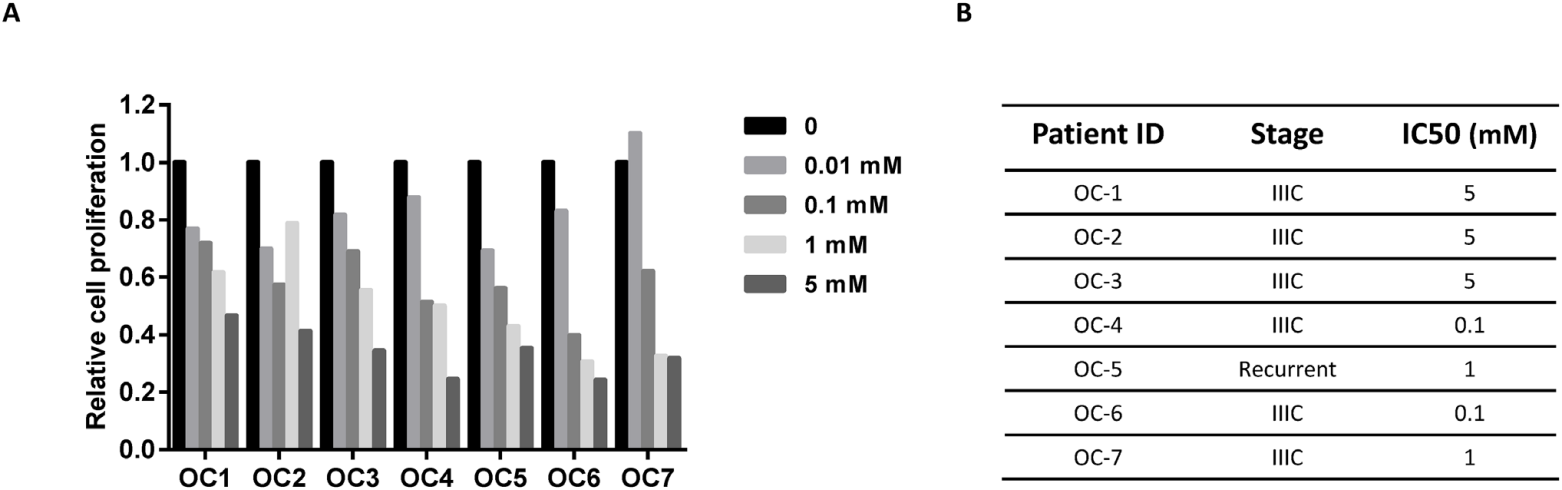
Phenformin inhibited cell proliferation in primary cultures of human OC **(A)** Cell proliferation was assayed by MTT assay in seven primary cultures of human OCs after 72 hours of treatment with phenformin. **(B)** The IC50 value and clinical characteristics for the OC primary cultures.

Primary cancer cell cultures have a greater capacity to predict realistic drug sensitivity than immortalized cancer cell lines. Thus, we further investigated the effects of phenformin on cell growth in primary cultures of OC cells using the MTT assay. Seven total primary cell cultures of OC were treated with different concentrations of phenformin for 72 hours. The results demonstrated that all seven primary cell cultures responded to phenformin and had achievable IC50 values (7/7, p=0.00001-0.015, IC50 range of 0.1 – 5 mM, Figure 7A and 7B).

## DISCUSSION

Multiple pre-clinical studies have demonstrated that the metformin has anti-neoplastic properties for a number of different cancers, including OC [24, 50–54]. Recent studies have also found that phenformin, another biguanide, may behave similarly to metformin in the inhibition of cell and tumor growth but with potentially heightened efficacy, due to its non-dependence on organic cation transporters for entry into cells [40]. The anti-tumorigenic activity of phenformin was first described in animal models in 1980 [45], and phenformin has since been found to have anti-neoplastic effects in breast, lung, prostate and colon cancers, among others. Phenformin has been shown to inhibit cellular proliferation [42, 55], induce apoptosis [43, 46], decrease cell cycle progression through increased expression of the cell cycle inhibitor p21 [55], suppress tumor growth and development *in vivo* [40, 41, 44–46], activate AMPK and inhibit the mTOR pathway [40, 43], inhibit the mesenchymal-epithelial transition [56] as well as tumor migration [56] and angiogenesis [43]. Most importantly, phenformin has been shown to be more effective when compared to metformin in the inhibition of tumor development and growth in colon cancer cell lines [42], leukemia cell lines [57], and breast cancer cell lines and mouse models, without increased murine toxicity [41, 43, 56, 58]. To date, the potential utility of phenformin in OC has not been well-studied.

We investigated the anti-neoplastic activity of phenformin in human OC cells, primary OC cultures and an orthotopic mouse model of high grade serous OC. Phenformin was found to inhibit cellular proliferation in a dose-dependent manner in all three human OC cell lines tested. It also exhibited more potent inhibition of cell proliferation and showed an enhanced affinity OCT1 and OCT3 compared to metformin in OC cells. We demonstrated that induction of apoptosis, cellular stress and cell cycle G1 arrest were key components of the anti-proliferative effects of phenformin in human OC cells, as evidenced by induced expression of annexin V and production of ROS as well as reduction of cyclin D and CDK4 expression. Phenformin was also found to inhibit adhesion and invasion in OC cells and reduced serum VEGF levels in M909 mice. Our results support previous results seen in other cancers including breast, colon and prostate [43, 46, 55, 56]. In addition, phenformin treatment (2mg/kg/day, intraperitoneal) led to profound inhibition of ovarian tumor growth *in vivo*. Our data supports prior studies that demonstate an anti-tumorigenic effect of phenformin *in vitro* and *in vivo* [40–46, 55–58].

Although biguanides have been shown to present anti-tumorigenic activity in many types of cancers including ovarian cancer, the detailed mechanisms of phenformin action against tumor have not been entirely defined. Several possible mechanisms related to the anti-tumorigenic activity of biguanides could be explained by activation of AMPK, which is in turn leading to the inhibition of mTOR signaling pathway. Our recent results showed that AMPK is an ideal target for the prevention and treatment of ovarian cancer. In this study, phenformin activated AMPK and inhibited the mTOR pathway as was demonstrated by increased phosphorylation of AMPK and decreased phosphorylation of S6 expression in all three OC cell lines. As seen in the OC cell lines, the phenformin-treated M909 ovarian tumors were shown to have decreased expression of Ki-67 and phosphorylated-S6 and an increase in phosphorylated AMPK and cleaved caspase 3. Thus, similar to metformin [12, 59], phenformin’s anti-tumorigenic effects *in vivo* may be through induction of cell cycle arrest and apoptosis via AMPK activation and inhibition of the downstream mTOR pathway.

AMPK activation by metformin is associated with increased oxidative stress leading to up-regulated cell cycle arrest and induction of apoptosis in breast cancer and leukemia [60, 61]. The anti-cancer effect of phenformin was related to complex I inhibition in the mitochondria and subsequent overproduction of reactive oxygen species (ROS) in colon cancer cells [47]. Treatment cancer cells with the ROS scavenger NAC significantly reduced the anti-cancer effect of phenformin, suggesting that anti-cancer action of phenformin is closely associated with the production of ROS [47]. In this study, a dose dependent increase in ROS levels with phenformin was observed by DCFH-DA fluorescent assay in ovarian cancer cell lines. Phenformin was also associated with increased the expression of PERK, Ero1-1α and PDI, which are markers of oxidative stress associated with apoptosis [61]. This is consistent with proposed mechanism of action of phenformin involving AMPK activation leading to mTOR inhibition in ovarian cancer cells.

AMPK signaling exerts regulatory effects on cancer cell adhesion, migration and invasion, which is involved in different mechanisms including disruption of the mTOR, TGF-b, Pdlim5, CXCL12, NF-κB and Akt-MDM2-Foxo3a pathways. Inhibition of melanoma cell invasion by metformin is correlated with modulation of expression of proteins involved in epithelial-mesenchymal transition such as Slug, Snail, SPARC, fibronectin, and N-cadherin and with inhibition of MMP-2 and MMP-9 activation [62]. We recently found that buformin significantly inhibits cell adhesion and invasion in endometrial cancer cells [63] and NT1014, a novel biguanide and AMPK activator, is able to reduce serum VEGF production and MMP expression in ovarian cancer tissues in a transgenic mouse model of ovarian cancer [50]. The current study further confirms that phenformin inhibits cell adhesion and invasion development through mediating the expression of epithelial–mesenchymal transition proteins and reducing VEGF and MMP productions in ovarian cancer. These findings bring new evidences to the understanding the antimetastatic effects of biguanides on ovarian cancer.

A major concern with phenformin is the increased risk of lactic acidosis seen in phenformin compared to metformin when used for the treatment of diabetes [64, 65]. In our study, we did not observe any overt signs of toxicity or lactic acidosis in the M909 mice treated with phenformin. Lactic acid is formed because biguanides impair mitochondrial respiration via inhibition of complex I, which can result in a compensatory acceleration of glycolysis to counteract the reduced ATP production via oxidative phosphorylation [66]. Metformin is a less powerful inhibitor of the mitochondrial respiratory chain which may decrease its risk for lactic acidosis [67]. The increased incidence of lactic acidosis in phenformin may also occur because phenformin inhibits lactate oxidation [68, 69] and increases the release of lactate from muscle while metformin increases lactate oxidation and does not alter the release of lactate from muscle [70]. However, since phenformin is safer and has fewer side effects than many of the typical cytotoxic and targeted agents used for OC treatment, this potential risk may be tolerated if phenformin has greater anti-neoplastic properties than metformin. Other common side effects of phenformin, as for metformin, are symptoms of gastrointestinal distress, such as nausea and diarrhea; however, the incidence of gastrointestinal side effects are more common with metformin than phenformin [71].

Given that biguanides are cleared through renal secretion, nearly all episodes of lactic acidosis associated with biguanides have occurred in patients with renal dysfunction [72]. Careful patient selection and observation may allow this side effect to be minimized. Moreover, treating cells with a combination of phenformin and 2-deoxyglucose or a lactate dehydrogenase (LDH) inhibitor, has been found to avoid the development of lactic acidosis [73]. In addition to reduction of lactic acidosis, combination treatment with phenformin and a LDH inhibitor led to increased inhibition of cell proliferation [42, 73, 74], suggesting that this may be a novel cancer treatment strategy for increased efficacy and reduced toxicity of phenformin.

One of the criticisms of the *in vitro* studies of metformin in cancer cell lines is that supra-physiological doses are needed to show an anti-proliferative effect, although clinically relevant doses have successfully inhibited tumor growth in animal cancer models [52, 75]. This was thought to be related to the relatively low expression of OCT 1 and 3 in solid tumors and cancer cell lines [76]. Given that phenformin is more lipophilic and does not need these transporter proteins to enter cells, it has been hypothesized that phenformin may be an improvement over metformin, in that it will have the same beneficial metabolic effects and potentially be better taken up by tumor cells. However, our *in vitro* studies as well as those of others [42–44, 46, 56, 58] still required supra-physiological doses of this drug in culture to achieve IC50 growth inhibition. Thus, the differences in the low expression of OCT 1 and 3 does not explain the increased dose needed to demonstrate an effect *in vitro*, and alternatively, this may be related to the hyperglycemic concentrations typically used to maintain and grow cancer cell lines in culture. In support of this, lower and more physiological concentrations of both metformin and phenformin have been found to decrease OC cell proliferation under normaglycemic *versus* hyperglycemic conditions [30]. For our M909 orthotopic OC mouse model, a clinically relevant dose for phenformin (2mg/kg/day, intraperitoneal) was used and proved to be effective in the inhibition of tumor growth.

In summary, the results from this study show that phenformin is a novel metabolic therapy that causes significant inhibition of OC cell proliferation *in vitro* and has anti-tumorigenic activity *in vivo* against OC. Given the increased risk of lactic acidosis, the risk/benefit ratio clearly favors metformin for the treatment of diabetes; however, this may not hold true for the treatment of cancer if phenformin was found to have superior anti-tumorigenic activity. Thus, our future work will entail a head-to-head comparison of phenformin against metformin in our orthotopic mouse model of serous OC. Lastly, an unanswered but important question in regards to phenformin and other biguanide treatment is whether these agents will be efficacious in all OC patients or more specifically beneficial in those that are obese or diabetic, similar to what has also been shown in breast and lung cancer mouse models [52, 77] and our own work in OC [78]. Given this, we plan to study the efficacy of phenformin *versus* metformin in our orthotopic OC mouse model under obese and lean conditions. Interestingly, metformin treatment has been previously found to elicit greater reductions in tumor growth in normoglycemic *versus* hyperglycemic conditions in a syngeneic OC mouse model [30], suggesting that diet-induced obesity *versus* hyperglycemia are not interchangeable and instead are distinct in their impact on metformin response. A better understanding of the interplay between the metabolic milieu and biguanide treatment is critically needed for the further development of these drugs in the treatment and prevention of all cancers, including OC.

## MATERIALS AND METHODS

### Cell culture and reagents

Three OC cell lines, SKOV3, Hey and IGROV-1, were used for the experiments. The SKOV3 cells were maintained in DMEM/F12 medium with 10% fetal bovine serum (FBS). The IGROV-1 cells were maintained in RPMI 1640 with 10% FBS. The Hey cell line was maintained in RPMI 1640 with 5% FBS. All medium was supplemented with 100 U/ml of penicillin and 100 ug/ml of streptomycin. The cells were cultured in a humidified 5% CO2 at 37°C. Phenformin was purchased from Sigma (St. Louis, MO). MTT (3-(,5-dimethylthiazol-2-yl)-2,5-diphenyltetrazolium bromide), RNase A and RIPA buffer were purchased from Sigma (St. Louis, MO). Antibodies to phosphorylated-AMPK (Thr172), phosphorylated-S6 (Ser 235/236), β-actin, pan-AMPK and pan-S6 were obtained from Cell Signaling Technology (Beverly, MA). The Annexin V FITC kit was purchased from BioVision (Mountain View, CA). Enhanced chemiluminescence Western blotting detection reagents were purchased from Amersham (Arlington Heights, IL). All other chemicals were purchased from Sigma.

### Cell proliferation assays

The SKOV3, Hey and IGROV-1 cells were plated and grown in 96-well plates at a concentration of 4000 cells/well for 24 hours. The cells were then treated with various concentrations of phenformin for a period of 72 hours. After the addition of MTT dye (5 mg/ml), the 96-well plates were incubated for an additional hour at 37°C. The MTT reaction was terminated through the addition of 100 uL of DMSO. The plates were read by measuring absorption at 595 nm with a Microplate Reader (Tecan, Morrisville, NC). The effect of phenformin was calculated as a percentage of control cell growth obtained from PBS (1%) treated cells grown in the same 96-well plates. Each experiment was performed in triplicate to assess for consistency of results.

### Cell cycle assay

The effect of phenformin on cell cycle progression was assessed using Cellometer (Nexcelom, Lawrence, MA). Cells were plated at a density of 2.5 x10^5^ cells/well in 6-well plates overnight and then treated with varying concentrations of phenformin for 24 hours. Cells were collected by 0.05% trypsin (Gibco, Grand Island, NY), washed with phosphate-buffered saline (PBS) solution, fixed in a 90% methanol solution and then stored at -20°C until cell cycle analysis was performed. On the day of analysis, the cells were washed with PBS and centrifuged, re-suspended in 50 ul RNase A solution (250 ug/ml) with 10 mM EDTA, followed by incubation for 30 min at 37°C. After incubation, 50 μl of propidium iodide (PI) staining solution (2 mg/ml PI, 0.1 mg/ml Azide and 0.05% Triton X-100) was added to each tube, and the cells were incubated for 10 min in the dark. The cells were then assessed by Cellometer. The results were analyzed using FCS4 express software (Molecular Devices, Sunnyvale, CA). Each experiment was performed in triplicate to assess for consistency of results.

### Apoptosis assay

The effect of phenformin on cell apoptosis was evaluated by using the Annexin-V FITC Apoptosis Detection Kit. The cells were plated in 6 well plates (2.5 x10^5^ cells/well) for 24 hours and then treated with varying concentrations of phenformin. The cells were collected by 0.25% trypsin without EDTA. After washing with PBS, cells were resuspended in 100 ul of Annexin-V and PI dual-stain solution (0.1 ug of Annexin-V FITC and 1 ug of PI) for 15 min in the dark. Apoptotic cells were detected by Cellometer. The results were analyzed by FCS4 express software. All experiments were performed in triplicate to assess for consistency of response.

### Adhesion assay

Each well in a 96-well plate was coated with 100 ul laminin-1 (10 ug/ml) and incubated at 37°C for 1 hour. This fluid was then aspirated, and 200ul blocking buffer was added to each well for 45-60 min at 37°C. The wells were then washed with PBS, and the plate was allowed to chill on ice. To each well, 2.5 x 10^3^ cells were added with PBS and varying concentrations of phenformin directly. The plate was then allowed to incubate at 37°C for 2 hours. After this period, the medium was aspirated, and cells were fixed by directly adding 100 ul of 5% glutaraldehyde and incubating for 30 min at room temperature. Adhered cells were then washed with PBS and stained with 100 ul of 0.1% crystal violet for 30 minutes. The cells were then washed repeatedly with water, and 100 ul of 10% acetic acid was added to each well to solubilize the dye. After 5 minutes of shaking, the absorbance was measured at 570 nm using a micro-plate reader from Tecan (Morrisville, NC). Each experiment was repeated at least twice for consistency of response.

### Invasion assay

Cell invasion assays were performed using 96-well HTS transwells (Corning Life Sciences, Durham, NC) coated with 0.5-1X BME (Trevigen, Gaithersburg, MD). The SKOV3, Hey and IGROV-1 (50,000/well) were starved for 12 hours and then seeded in the upper chambers of the wells in 50 μl FBS-free medium. The lower chambers were filled with 150 μl regular medium with phenformin. The plate was incubated for 24 hours at 37°C to allow invasion into the lower chamber. After washing the upper and lower chambers with PBS, 100 ul Calcein AM solution was added into the lower chamber and incubated at 37°C for 30-60 min. The lower chamber plate was measured by the plate reader for reading fluorescence at EX/EM 485/520 nM. Each experiment was repeated at least twice for consistency of response.

### Western immunoblotting

The SKOV3, Hey, and IGROV-1 cells were plated at 2.5 x10^5^ cells/well in 6 well plates in their appropriate media and were treated for 24 hours with phenformin. Cell lysates were prepared in RIPA buffer (1% NP40, 0.5 sodium deoxycholate and 0.1% SDS) plus PhosStop. Equal amounts of protein were separated by gel electrophoresis and transferred onto a PVDF membrane. The membrane was blocked with 5% nonfat dry milk and then incubated with a 1:1000 dilution of primary antibody overnight at 4°C. The membrane was then washed and incubated with a secondary peroxidase conjugated antibody for 1 hour after washing. Antibody binding was detected using an enhanced chemiluminescence detection buffer and the Alpha Innotech Imaging System (San Leandro, CA). After developing, the membrane was re-probed using antibody against α-tubulin or β-actin as a control for equal loading. Each experiment was repeated three times to assess for consistency of results.

### Measurement of VEGF levels in serum

The VEGF concentration in the serum of mice after exposure to phenformin was measured with a VEGF ELISA kit (DVE00, R&D Systems, Minneapolis, MN), according to the manufacturer’s instructions. 10 ul of serum from phenformin and control groups (6 samples /per group) was used in the assay. The optical density at 570 nm of each well was measured using a FLUOstar OMEGA reader (Cary, NC).

### Reactive oxygen species (ROS) assay

The alteration of total production of reactive oxygen species caused by phenformin was measured using a DCFH-DA fluorescent dye. The SKOV3, Hey and IGROV-1 cells (1.0 × 10^4^ cells/well) were seeded in black 96-well plates. After 24 hours, the cells were treated with phenformin (0.1 to 5 mM) for 4 hours to induce ROS generation. After the cells were incubated with DCFH-DA (20 μM) for 30 min, the fluorescence was monitored at an excitation wavelength of 485 nm and an emission wavelength of 530 nm using a plate reader (Tecan). All experiments were performed at least twice to assess for consistency of response.

### Primary OC cell culture

Seven tumor specimens were sampled from patients undergoing surgery for high grade serous OC at an academic institution. The protocol was reviewed and approval granted by the Institutional Review Board. Freshly obtained tissues were washed three times with Hank's Buffered Salt Solution and then gently minced by scissors in DMEM/F12 medium containing 10% FBS. These tissues were then digested in 0.2% collagenase IA, 100 U/ml penicillin and anti-anti for 30-60 min in a 37°C water bath with shaking. After two centrifugations with PBS solution, cells were re-suspended and diluted to 1x10^5^ cells/ml with DMEM/F12 medium. 2x10^4^ cells/well were seeded into 96-well plates and incubated for 24 hours before treatment with phenformin. Cell proliferation was measured by MTT assay after 72 hours of phenformin treatment.

### Orthotropic xenografts of serous OC

The K18-gT121^+/-^p53 ^fl/fl^Brca1 ^fl/fl^ (KpB) mouse model is a high grade serous OC mouse model that specifically and somatically deletes the tumor suppressor genes, Brca1 and p53, and inactivates the retinoblastoma (Rb) proteins in adult ovarian surface epithelial cells (KpB mouse model) [48]. As an extension of this model, we have established an ovarian tumor cell line from one of the KpB mice (M909). Upon re-injection of these tumor cells into the ovarian bursa of female mice, we have developed a more aggressive variant of the KpB model [79]. For the evaluation of phenformin’s *in vivo* effects, M909 cells (1X10^6^ cells/5 μl) were injected into the left side of the ovarian bursa of 6–8 week old mice. All mice were handled according to protocols approved by the Institutional Animal Care and Use Committee (IACUC). Twenty-six mice were injected with the M909 cells and then randomly divided into the vehicle (i.e. control) or phenformin group. Phenformin (2 mg/kg/day, intraperitoneal) and vehicle treatment was initiated after palpation of a 0.1 cm tumor in the mice. Tumor size was checked twice a week by caliper measurement. Tumor volume was calculated using the following equation (width^2^ × length)/2. All mice were euthanized after four weeks of phenformin or vehicle treatment. At sacrifice, mice were weighed and blood samples were taken. Half of the ovarian tumor was snap-frozen and stored at −80°C, and the other half was fixed in 10% neutral-buffered formalin and paraffin embedded.

### Immunohistochemistry

Five micrometer paraffin sections, prepared from the ovarian tumors of the M909-injected mice, were used for immunohistochemical analysis. Staining procedures were performed at the IHC Mice Core Facility. The following primary antibodies were used: Ki-67, cleaved caspase-3, MMP9, phosphorylated-AMPK and phosphorylated-S6. Further processing was carried out using the ABC-Staining Kits (Vector Labs, Burlingame, CA) and hematoxylin. Immunochemistry slides were scanned, analyzed and scored by Aperio and ImageScope software (Vista, CA).

### Statistical analysis

Data were presented as mean ± standard error of the mean. Statistical analysis of the differences between the groups was determined with the two-sided unpaired Student’s t-test using GraphPad software (La Jolla, CA USA). A value of p<0.05 was considered significant.

## References

[1] Jang M, Kim SS, Lee J (2013). Cancer cell metabolism: implications for therapeutic targets. Experimental & molecular medicine.

[2] Delort L, Kwiatkowski F, Chalabi N, Satih S, Bignon YJ, Bernard-Gallon DJ (2009). Central adiposity as a major risk factor of ovarian cancer. Anticancer Res.

[3] Olsen CM, Nagle CM, Whiteman DC, Ness R, Pearce CL, Pike MC, Rossing MA, Terry KL, Wu AH, Risch HA, Yu H, Australian Ovarian Cancer Study Group (2013). Obesity and risk of ovarian cancer subtypes: evidence from the Ovarian Cancer Association Consortium. Endocr Relat Cancer.

[4] Leitzmann MF, Koebnick C, Danforth KN, Brinton LA, Moore SC, Hollenbeck AR, Schatzkin A, Lacey JV (2009). Body mass index and risk of ovarian cancer. Cancer.

[5] Guh DP, Zhang W, Bansback N, Amarsi Z, Birmingham CL, Anis AH (2009). The incidence of co-morbidities related to obesity and overweight: a systematic review and meta-analysis. BMC Public Health.

[6] Lahmann PH, Cust AE, Friedenreich CM, Schulz M, Lukanova A, Kaaks R, Lundin E, Tjonneland A, Halkjaer J, Severinsen MT, Overvad K, Fournier A, Chabbert-Buffet N (2010). Anthropometric measures and epithelial ovarian cancer risk in the European Prospective Investigation into Cancer and Nutrition. Int J Cancer.

[7] Yang HS, Yoon C, Myung SK, Park SM (2011). Effect of obesity on survival of women with epithelial ovarian cancer: a systematic review and meta-analysis of observational studies. Int J Gynecol Cancer.

[8] Reeves GK, Pirie K, Beral V, Green J, Spencer E, Bull D, Million Women Study Collaboration (2007). Cancer incidence and mortality in relation to body mass index in the Million Women Study: cohort study. BMJ.

[9] Engeland A, Tretli S, Bjorge T (2003). Height, body mass index, and ovarian cancer: a follow-up of 1.1 million Norwegian women. J Natl Cancer Inst.

[10] National Health Examination Survey Data (2012).

[11] Bakhru A, Buckanovich RJ, Griggs JJ (2011). The impact of diabetes on survival in women with ovarian cancer. Gynecologic oncology.

[12] Stine JE, Bae-Jump V (2014). Metformin and gynecologic cancers. Obstetrical & gynecological survey.

[13] van Dam PA, Vergote IB, Lowe DG, Watson JV, van Damme P, van der Auwera JC, Shepherd JH (1994). Expression of c-erbB-2, c-myc, and c-ras oncoproteins, insulin-like growth factor receptor I, and epidermal growth factor receptor in ovarian carcinoma. J Clin Pathol.

[14] Brokaw J, Katsaros D, Wiley A, Lu L, Su D, Sochirca O, de la Longrais IA, Mayne S, Risch H, Yu H (2007). IGF-I in epithelial ovarian cancer and its role in disease progression. Growth Factors.

[15] Spentzos D, Cannistra SA, Grall F, Levine DA, Pillay K, Libermann TA, Mantzoros CS (2007). IGF axis gene expression patterns are prognostic of survival in epithelial ovarian cancer. Endocr Relat Cancer.

[16] Levine DA, Bogomolniy F, Yee CJ, Lash A, Barakat RR, Borgen PI, Boyd J (2005). Frequent mutation of the PIK3CA gene in ovarian and breast cancers. Clin Cancer Res.

[17] Bast RC, Mills GB (2012). Dissecting “PI3Kness“: the complexity of personalized therapy for ovarian cancer. Cancer discovery.

[18] Makowski L, Zhou C, Zhong Y, Kuan PF, Fan C, Sampey BP, Difurio M, Bae-Jump VL (2014). Obesity increases tumor aggressiveness in a genetically engineered mouse model of serous ovarian cancer. Gynecol Oncol.

[19] Evans JM, Donnelly LA, Emslie-Smith AM, Alessi DR, Morris AD (2005). Metformin and reduced risk of cancer in diabetic patients. Bmj.

[20] Bowker SL, Majumdar SR, Veugelers P, Johnson JA (2006). Increased cancer-related mortality for patients with type 2 diabetes who use sulfonylureas or insulin. Diabetes Care.

[21] Libby G, Donnelly LA, Donnan PT, Alessi DR, Morris AD, Evans JM (2009). New users of metformin are at low risk of incident cancer: a cohort study among people with type 2 diabetes. Diabetes Care.

[22] Bodmer M, Becker C, Meier C, Jick SS, Meier CR (2011). Use of metformin and the risk of ovarian cancer: a case-control analysis. Gynecol Oncol.

[23] Currie CJ, Poole CD, Jenkins-Jones S, Gale EA, Johnson JA, Morgan CL (2012). Mortality after incident cancer in people with and without type 2 diabetes: impact of metformin on survival. Diabetes Care.

[24] Romero IL, McCormick A, McEwen KA, Park S, Karrison T, Yamada SD, Pannain S, Lengyel E (2012). Relationship of type II diabetes and metformin use to ovarian cancer progression, survival, and chemosensitivity. Obstetrics and gynecology.

[25] Kumar S, Meuter A, Thapa P, Langstraat C, Giri S, Chien J, Rattan R, Cliby W, Shridhar V (2013). Metformin intake is associated with better survival in ovarian cancer: a case-control study. Cancer.

[26] Dilokthornsakul P, Chaiyakunapruk N, Termrungruanglert W, Pratoomsoot C, Saokaew S, Sruamsiri R (2013). The effects of metformin on ovarian cancer: a systematic review. Int J Gynecol Cancer.

[27] Yasmeen A, Beauchamp MC, Piura E, Segal E, Pollak M, Gotlieb WH (2011). Induction of apoptosis by metformin in epithelial ovarian cancer: involvement of the Bcl-2 family proteins. Gynecol Oncol.

[28] Liao H, Zhou Q, Gu Y, Duan T, Feng Y (2012). Luteinizing hormone facilitates angiogenesis in ovarian epithelial tumor cells and metformin inhibits the effect through the mTOR signaling pathway. Oncol Rep.

[29] Gwak H, Kim Y, An H, Dhanasekaran DN, Song YS (2016). Metformin induces degradation of cyclin D1 via AMPK/GSK3beta axis in ovarian cancer. Mol Carcinog.

[30] Litchfield LM, Mukherjee A, Eckert MA, Johnson A, Mills KA, Pan S, Shridhar V, Lengyel E, Romero IL (2015). Hyperglycemia-induced metabolic compensation inhibits metformin sensitivity in ovarian cancer. Oncotarget.

[31] Rattan R, Graham RP, Maguire JL, Giri S, Shridhar V (2011). Metformin suppresses ovarian cancer growth and metastasis with enhancement of cisplatin cytotoxicity *in vivo*. Neoplasia.

[32] Shank JJ, Yang K, Ghannam J, Cabrera L, Johnston CJ, Reynolds RK, Buckanovich RJ (2012). Metformin targets ovarian cancer stem cells *in vitro* and *in vivo*. Gynecol Oncol.

[33] Lengyel E, Litchfield LM, Mitra AK, Nieman KM, Mukherjee A, Zhang Y, Johnson A, Bradaric M, Lee W, Romero IL (2015). Metformin inhibits ovarian cancer growth and increases sensitivity to paclitaxel in mouse models. Am J Obstet Gynecol.

[34] Tebbe C, Chhina J, Dar SA, Sarigiannis K, Giri S, Munkarah AR, Rattan R (2014). Metformin limits the adipocyte tumor-promoting effect on ovarian cancer. Oncotarget.

[35] Erices R, Bravo ML, Gonzalez P, Oliva B, Racordon D, Garrido M, Ibanez C, Kato S, Branes J, Pizarro J, Barriga MI, Barra A, Bravo E (2013). Metformin, at concentrations corresponding to the treatment of diabetes, potentiates the cytotoxic effects of carboplatin in cultures of ovarian cancer cells. Reprod Sci.

[36] Galdieri L, Gatla H, Vancurova I, Vancura A (2016). Activation of AMP-Activated Protein Kinase by Metformin Induces Protein Acetylation in Prostate and Ovarian Cancer Cells. J Biol Chem.

[37] Hynninen P, Vaskivuo L, Saarnio J, Haapasalo H, Kivela J, Pastorekova S, Pastorek J, Waheed A, Sly WS, Puistola U, Parkkila S (2006). Expression of transmembrane carbonic anhydrases IX and XII in ovarian tumours. Histopathology.

[38] McGuinness ME, Talbert RL (1993). Phenformin-induced lactic acidosis: a forgotten adverse drug reaction. Ann Pharmacother.

[39] Graham GG, Punt J, Arora M, Day RO, Doogue MP, Duong JK, Furlong TJ, Greenfield JR, Greenup LC, Kirkpatrick CM, Ray JE, Timmins P, Williams KM (2011). Clinical pharmacokinetics of metformin. Clinical pharmacokinetics.

[40] Huang X, Wullschleger S, Shpiro N, McGuire VA, Sakamoto K, Woods YL, McBurnie W, Fleming S, Alessi DR (2008). Important role of the LKB1-AMPK pathway in suppressing tumorigenesis in PTEN-deficient mice. The Biochemical journal.

[41] Appleyard MV, Murray KE, Coates PJ, Wullschleger S, Bray SE, Kernohan NM, Fleming S, Alessi DR, Thompson AM (2012). Phenformin as prophylaxis and therapy in breast cancer xenografts. Br J Cancer.

[42] Lea MA, Chacko J, Bolikal S, Hong JY, Chung R, Ortega A, desbordes C (2011). Addition of 2-deoxyglucose enhances growth inhibition but reverses acidification in colon cancer cells treated with phenformin. Anticancer Res.

[43] Orecchioni S, Reggiani F, Talarico G, Mancuso P, Calleri A, Gregato G, Labanca V, Noonan DM, Dallaglio K, Albini A, Bertolini F (2015). The biguanides metformin and phenformin inhibit angiogenesis, local and metastatic growth of breast cancer by targeting both neoplastic and microenvironment cells. Int J Cancer.

[44] Jiang W, Finniss S, Cazacu S, Xiang C, Brodie Z, Mikkelsen T, Poisson L, Shackelford DB, Brodie C (2016). Repurposing phenformin for the targeting of glioma stem cells and the treatment of glioblastoma. Oncotarget.

[45] Dilman VM, Anisimov VN (1980). Effect of treatment with phenformin, diphenylhydantoin or L-dopa on life span and tumour incidence in C3H/Sn mice. Gerontology.

[46] Shackelford DB, Abt E, Gerken L, Vasquez DS, Seki A, Leblanc M, Wei L, Fishbein MC, Czernin J, Mischel PS, Shaw RJ (2013). LKB1 inactivation dictates therapeutic response of non-small cell lung cancer to the metabolism drug phenformin. Cancer cell.

[47] Miskimins WK, Ahn HJ, Kim JY, Ryu S, Jung YS, Choi JY (2014). Synergistic anti-cancer effect of phenformin and oxamate. PloS one.

[48] Szabova L, Yin C, Bupp S, Guerin TM, Schlomer JJ, Householder DB, Baran ML, Yi M, Song Y, Sun W, McDunn JE, Martin PL, Van Dyke T, Difilippantonio S (2012). Perturbation of Rb, p53, and Brca1 or Brca2 cooperate in inducing metastatic serous epithelial ovarian cancer. Cancer research.

[49] Jefferies HB, Fumagalli S, Dennis PB, Reinhard C, Pearson RB, Thomas G (1997). Rapamycin suppresses 5'TOP mRNA translation through inhibition of p70s6k. Embo J.

[50] Zhang L, Han J, Jackson AL, Clark LN, Kilgore J, Guo H, Livingston N, Batchelor K, Yin Y, Gilliam TP, Gehrig PA, Sheng X, Zhou C, Bae-Jump VL (2016). NT1014, a novel biguanide, inhibits ovarian cancer growth *in vitro* and *in vivo*. Journal of hematology & oncology.

[51] Dowling RJ, Zakikhani M, Fantus IG, Pollak M, Sonenberg N (2007). Metformin inhibits mammalian target of rapamycin-dependent translation initiation in breast cancer cells. Cancer Res.

[52] Algire C, Amrein L, Zakikhani M, Panasci L, Pollak M (2010). Metformin blocks the stimulative effect of a high-energy diet on colon carcinoma growth *in vivo* and is associated with reduced expression of fatty acid synthase. Endocr Relat Cancer.

[53] Zakikhani M, Dowling RJ, Sonenberg N, Pollak MN (2008). The effects of adiponectin and metformin on prostate and colon neoplasia involve activation of AMP-activated protein kinase. Cancer Prev Res (Phila).

[54] Anisimov VN, Egormin PA, Bershtein LM, Zabezhinskii MA, Piskunova TS, Popovich IG, Semenchenko AV (2005). Metformin decelerates aging and development of mammary tumors in HER-2/neu transgenic mice. Bull Exp Biol Med.

[55] Caraci F, Chisari M, Frasca G, Chiechio S, Salomone S, Pinto A, Sortino MA, Bianchi A (2003). Effects of phenformin on the proliferation of human tumor cell lines. Life Sci.

[56] Liu Z, Ren L, Liu C, Xia T, Zha X, Wang S (2015). Phenformin Induces Cell Cycle Change, Apoptosis, and Mesenchymal-Epithelial Transition and Regulates the AMPK/mTOR/p70s6k and MAPK/ERK Pathways in Breast Cancer Cells. PloS one.

[57] Velez J, Pan R, Lee JT, Enciso L, Suarez M, Duque JE, Jaramillo D, Lopez C, Morales L, Bornmann W, Konopleva M, Krystal G, Andreeff M, Samudio I (2016). Biguanides sensitize leukemia cells to ABT-737-induced apoptosis by inhibiting mitochondrial electron transport. Oncotarget.

[58] El-Masry OS, Brown BL, Dobson PR (2012). Effects of activation of AMPK on human breast cancer cell lines with different genetic backgrounds. Oncol Lett.

[59] Gadducci A, Biglia N, Tana R, Cosio S, Gallo M (2016). Metformin use and gynecological cancers: A novel treatment option emerging from drug repositioning. Crit Rev Oncol Hematol.

[60] Queiroz EA, Puukila S, Eichler R, Sampaio SC, Forsyth HL, Lees SJ, Barbosa AM, Dekker RF, Fortes ZB, Khaper N (2014). Metformin induces apoptosis and cell cycle arrest mediated by oxidative stress, AMPK and FOXO3a in MCF-7 breast cancer cells. PloS one.

[61] Leclerc GM, Leclerc GJ, Kuznetsov JN, DeSalvo J, Barredo JC (2013). Metformin induces apoptosis through AMPK-dependent inhibition of UPR signaling in ALL lymphoblasts. PloS one.

[62] Cerezo M, Tichet M, Abbe P, Ohanna M, Lehraiki A, Rouaud F, Allegra M, Giacchero D, Bahadoran P, Bertolotto C, Tartare-Deckert S, Ballotti R, Rocchi S (2013). Metformin blocks melanoma invasion and metastasis development in AMPK/p53-dependent manner. Molecular cancer therapeutics.

[63] Kilgore J, Jackson AL, Clark LH, Guo H, Zhang L, Jones HM, Gilliam TP, Gehrig PA, Zhou C, Bae-Jump VL (2016). Buformin exhibits anti-proliferative and anti-invasive effects in endometrial cancer cells. American journal of translational research.

[64] Dembo AJ, Marliss EB, Halperin ML (1975). Insulin therapy in phenformin-associated lactic acidosis; a case report, biochemical considerations and review of the literature. Diabetes.

[65] Kwong SC, Brubacher J (1998). Phenformin and lactic acidosis: a case report and review. J Emerg Med.

[66] Owen MR, Doran E, Halestrap AP (2000). Evidence that metformin exerts its anti-diabetic effects through inhibition of complex 1 of the mitochondrial respiratory chain. The Biochemical journal.

[67] Pernicova I, Korbonits M (2014). Metformin--mode of action and clinical implications for diabetes and cancer. Nature reviews Endocrinology.

[68] Searle GL, Siperstein MD (1975). Lactic acidosis associated with phenformin therapy. Evidence that inhibited lactate oxidation is the causative factor. Diabetes.

[69] Williams RH, Steiner DF (1959). Summarization of studies relative to the mechanism of phenethylbiguanide hypoglycemia. Metabolism.

[70] Stumvoll M, Nurjhan N, Perriello G, Dailey G, Gerich JE (1995). Metabolic effects of metformin in non-insulin-dependent diabetes mellitus. N Engl J Med.

[71] Cairns SA, Shalet S, Marshall AJ, Hartog M (1977). A comparison of phenformin and metformin in the treatment of maturity onset diabetes. Diabete Metab.

[72] Choi YK, Park KG (2013). Metabolic roles of AMPK and metformin in cancer cells. Mol Cells.

[73] Lea MA, Guzman Y, Desbordes C (2016). Inhibition of Growth by Combined Treatment with Inhibitors of Lactate Dehydrogenase and either Phenformin or Inhibitors of 6-Phosphofructo-2-kinase/Fructose-2,6-bisphosphatase 3. Anticancer Res.

[74] Al-Wahab Z, Tebbe C, Chhina J, Dar SA, Morris RT, Ali-Fehmi R, Giri S, Munkarah AR, Rattan R (2014). Dietary energy balance modulates ovarian cancer progression and metastasis. Oncotarget.

[75] Suh DH, Kim JW, Kang S, Kim HJ, Lee KH (2014). Major clinical research advances in gynecologic cancer in 2013. Journal of gynecologic oncology.

[76] Segal ED, Yasmeen A, Beauchamp MC, Rosenblatt J, Pollak M, Gotlieb WH (2011). Relevance of the OCT1 transporter to the antineoplastic effect of biguanides. Biochemical and biophysical research communications.

[77] Phoenix KN, Vumbaca F, Fox MM, Evans R, Claffey KP (2009). Dietary energy availability affects primary and metastatic breast cancer and metformin efficacy. Breast Cancer Res Treat.

[78] Jackson AL, Zhong Y, Zhou C, Kilgore J, Makowski L, Gehrig PA, Bae-Jump VL (2014). Metformin has increased efficacy under obese conditions in a novel genetically engineered mouse model of serous ovarian cancer. Annual Meeting of the Society of Gynecologic Oncology Annual Meeting 2014. Gynecologic Oncology.

[79] Qiu H, Jackson AL, Kilgore JE, Zhong Y, Chan LL, Gehrig PA, Zhou C, Bae-Jump VL (2015). JQ1 suppresses tumor growth through downregulating LDHA in ovarian cancer. Oncotarget.

